# Beyond the average brain: individual differences in social brain development are associated with friendship quality

**DOI:** 10.1093/scan/nsaa166

**Published:** 2020-12-05

**Authors:** Andrik I Becht, Lara M Wierenga, Kathryn L Mills, Rosa Meuwese, Anna van Duijvenvoorde, Sarah-Jayne Blakemore, Berna Güroğlu, Eveline A Crone

**Affiliations:** Erasmus School of Social and Behavioural Sciences, Erasmus University Rotterdam, Rotterdam 3062PA, The Netherlands; Research Center Adolescent Development, Utrecht University, Utrecht 3584CS, The Netherlands; Brain and Development Research Center, Leiden University, Leiden 2333AK, The Netherlands; Brain and Development Research Center, Leiden University, Leiden 2333AK, The Netherlands; Department of Psychology, University of Oregon, Eugene, OR 97403, USA; Brain and Development Research Center, Leiden University, Leiden 2333AK, The Netherlands; Brain and Development Research Center, Leiden University, Leiden 2333AK, The Netherlands; Department of Psychology, University of Cambridge, Cambridge CB2 3EB, UK; Brain and Development Research Center, Leiden University, Leiden 2333AK, The Netherlands; Erasmus School of Social and Behavioural Sciences, Erasmus University Rotterdam, Rotterdam 3062PA, The Netherlands; Brain and Development Research Center, Leiden University, Leiden 2333AK, The Netherlands

**Keywords:** structural brain development, social brain, adolescence, friendship quality, longitudinal

## Abstract

We tested whether adolescents differ from each other in the structural development of the social brain and whether individual differences in social brain development predicted variability in friendship quality development. Adolescents (*N *= 299, *M*_age_ T1* *=_ _13.98 years) were followed across three biannual waves. We analysed self-reported friendship quality with the best friend at T1 and T3, and bilateral measures of surface area and cortical thickness of the medial prefrontal cortex (mPFC), posterior superior temporal sulcus (pSTS), temporoparietal junction (TPJ) and precuneus across all waves. At the group level, growth curve models confirmed non-linear decreases of surface area and cortical thickness in social brain regions. We identified substantial individual differences in levels and change rates of social brain regions, especially for surface area of the mPFC, pSTS and TPJ. Change rates of cortical thickness varied less between persons. Higher levels of mPFC surface area and cortical thickness predicted stronger increases in friendship quality over time. Moreover, faster cortical thinning of mPFC surface area predicted a stronger increase in friendship quality. Higher levels of TPJ cortical thickness predicted lower friendship quality. Together, our results indicate heterogeneity in social brain development and how this variability uniquely predicts friendship quality development.

An essential developmental task of adolescents is to form and maintain high-quality friendships (Brown, 2004). Yet, not all adolescents are equally successful in completing this developmental task, which increases their risk of developing adjustment problems such as depression (for a meta-analysis, see Rueger *et al.*, 2016) and low self-esteem (Gorrese and Ruggieri, 2013). Adolescents’ development of a network of brain regions, referred to as the social brain, is considered particularly important for social functioning (Burnett *et al.*, 2011; Blakemore, 2012; Blakemore and Mills, 2014). This social brain network includes the medial prefrontal cortex (mPFC), temporoparietal junction (TPJ), posterior superior temporal sulcus (pSTS) and precuneus (Blakemore, 2012; Mills *et al.*, 2014). Group-level studies revealed that the structure of the social brain continues to develop across adolescence (Mills *et al.*, 2014). Yet, no studies have tested whether adolescents show individual differences in the rate of change in social brain regions (Foulkes and Blakemore, 2018; Becht and Mills, 2020), despite an increasing interest to use information on individual differences in brain development to predict mental health outcomes (Rosenberg *et al.*, 2018). Therefore, it is of paramount importance to study individual differences in social brain development beyond the average trajectories to get a better understanding as to why some adolescents are able to develop high-quality friendships, whereas others do not.

A critical assumption is that some trajectories of change may be more malleable to environmental input than others (Noble *et al.*, 2015; Piccolo, Merz, He, Sowell, and Noble, 2016), which may be indicated by individual differences in the baseline and speed of brain maturation that define a certain window of opportunity (Crone and Elzinga, 2015). For instance, some developmental growth patterns may be more genetically influenced and show relative constant changes for all individuals over time, comparable to developmental milestones that occur approximately around the same age in all individuals (Teeuw *et al.*, 2019). In contrast, other trajectories may show larger between-individual differences in change rates that may be related to individual differences in the environment as well (van der Meulen *et al.*, 2020). Prior behavioural developmental studies have used structural equation modelling techniques that are developed to directly examine questions regarding individual differences in change rates and how these individual differences in change predict outcomes (Kline, 2015). For example, adolescents’ onset of alcohol use can be predicted by individual differences in the development of close friends’ norms regarding alcohol use (Janssen *et al.*, 2018). An important direction for research on structural brain development is to use this approach and test for variability in slope patterns over time and link these to behavioural variability (Foulkes and Blakemore, 2018; Becht and Mills, 2020). Therefore, the first aim of this study was to statistically test whether there were significant individual differences in within-subject change in social brain development from late childhood into young adulthood across three time points, which could then be used to predict individual differences in social functioning.

In case of individual differences in development, the question emerges—how these individual differences may be related to social development. Functional magnetic resonance imaging (MRI) studies have shown that social brain regions are consistently implicated in tasks that involve social-cognitive processes, such as mentalizing (representing one’s own and others’ mental states), which is a vital capacity to understand and interact with others (Frith and Frith, 2001; Burnett *et al.*, 2011). The development of high-quality peer relationships is considered an important outcome of adolescents’ social-cognitive functioning. Moreover, having high-quality friendships affects adolescents’ current and future social functioning (Berndt, 2002). However, how structural social brain development is associated with friendship quality is yet unknown. Our second aim was therefore to examine whether individual-level variability in social brain development predicts individual differences in the development of friendship quality. Based on a limited number of longitudinal structural magnetic resonance imaging (MRI) studies sMRI studies, we expected that individuals who show relatively faster rates of cortical thinning (i.e. reflecting accelerated brain maturation) would show the largest increase in friendship quality over time (Ferschmann *et al.*, 2018, 2019). For example, a study on personality and structural brain development showed that those adolescents with a more mature personality at the first wave (indicated by higher levels of conscientiousness, emotional stability and imagination) showed accelerated cortical thinning in different brain areas over time (Ferschmann *et al.*, 2018). Similarly, greater rates of cortical thinning in the mPFC, TPJ and pSTS were related to higher levels of prosocial behaviour during adolescence (Ferschmann *et al.*, 2019).

## Present study

In sum, the current study had two aims. First, we examined development of the social brain regions at the group level (i.e. mean level development across individuals) and individual differences of social brain development across adolescence. Based on prior longitudinal work, we predicted structural brain maturation, demonstrated by mean-level linear or curvilinear decreases in the structure (i.e. surface area and thickness) of social brain regions from late childhood into young adulthood (Mills *et al.*, 2014). Pertaining to our main aim, we tested for significant individual differences in the baseline (i.e. intercept) and rate of change (slope) across development across four social brain regions following Foulkes and Blakemore (2018). Second, we tested whether these individual differences in the baseline and rate of change in social brain regions predicted changes in friendship quality over time, following recent suggestions to predict relevant outcomes from individual differences in neurobiological trajectories (Rosenberg *et al.*, 2018). We predicted that those adolescents who showed advanced brain maturation (i.e. faster rates of cortical thinning) in social brain regions would show the strongest increase in friendship quality over time.

## Method

### Participants and procedure

Participants were 299 Dutch individuals (52% girls; *M*_age_ T1* *= 13.98 years, s.d.* *= 3.68, range T1 = 8.01–25.95 years) who participated in the accelerated longitudinal Braintime study. The Braintime study includes three assessment waves (T1–T3) that are separated by a 2-year interval (for a detailed description of the sample, see, e.g., Peters *et al.*, 2016; Becht *et al.*, 2018). Participants came to the lab for the scan session. They watched a movie of their own choice during the high-resolution scan, which was administrated at the end of the scan session. Participants received €30 (equivalent to US$33) for participation at each assessment wave. Written informed consent was obtained from all participants at each wave. When participants were below 18 years of age, we requested additional consent from their parents. All study procedures were approved by the local institutional review boards. All participants were right-handed and reported no neurological or psychiatric impairment at Wave 1.

Missing value analyses indicated that on average participants completed 77% of all possible data points across waves. Little’s (1988) missing completely at random (MCAR) test revealed a chi-square (χ^2^/df) of 1.06, demonstrating that it is unlikely that findings were biased as a result of missing values. Hence, missing data were handled in Mplus 8.2 using full information maximum likelihood.

### Measures

#### Friendship quality.

We assessed individuals’ quality of their best friend relationship using the Dutch and shortened version of the Friendship Quality Scale (Bukowski *et al.*, 1994). We used the positive quality subscale (13 items) to tap into key components of the best friend relationship such as the level of closeness, security and companionship. Items were rated on a 5-point Likert scale (1 = ‘not true at all’, 5 = ‘very true’). We computed a mean friendship quality score for each individual at T1 and T3. Reliability of this scale was good with Cronbach’s alpha of 0.83 at T1 and T3. See Supplementary Figure S1 for the histograms of the mean friendship quality scores at T1 and T3.

#### Neuroimaging measures.

All participants were scanned on the same 3T MRI scanner (Tesla, Philips Achieva MRI system Best, The Netherlands). Technical details and procedures of the anatomical scans as well as image processing can be found as online Supplementary material S1. Post-processing of the scan quality was conducted using a semi-automatic quality assessment tool (Klapwijk *et al.*, 2019). Our final dataset included 677 scans from 270 participants. One hundred and sixty-eight participants had usable scans at three waves, 71 participants had scans at two waves and 31 participants had scans at one wave.

#### Social brain ROIs.

For three of our four social brain regions of interest (ROIs: mPFC, TPJ and pSTS), we used the same templates as used and described in full detail by Mills *et al.* (2014) and van der Meulen *et al.* (2020). These templates are also available here: https://figshare.com/articles/Social_Brain_Freesurfer_ROIs/726133. These ROIs were defined based on Brodmann’s areas, the Desikan–Killiany atlas (Desikan *et al.*, 2006) and functional coordinates. The precuneus was derived from the Desikan–Killiany atlas (Desikan *et al.*, 2006). Due to poor scan quality of the temporal pole region, the development of the anterior temporal cortex could not be analysed. We examined the mPFC, TPJ, pSTS and precuneus in all our subsequent longitudinal analyses. We averaged all ROIs across hemispheres. The visualization of these ROIs is presented in prior work by van der Meulen *et al.* (2020).

### Statistical analyses

**Fig. 1.**
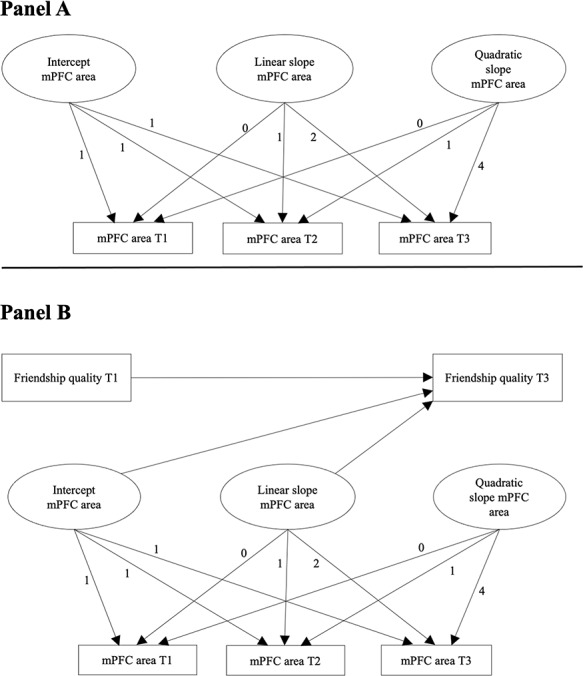
(A) Example of a latent growth curve model including an intercept, linear slope and quadratic slope to model the development of mPFC area across three waves. (B) Intercept, linear and quadratic slope of mPFC surface area predicting changes in friendship quality over time. mPFC = medial prefrontal cortex.

To examine development of the social brain at the group or mean level as well as individual differences in social brain development from childhood into emerging adulthood (Aim 1), we conducted a series of latent growth curve models (Duncan and Duncan, 2009). Specifically, we fitted a latent growth curve model (LGM) for each social brain region (i.e. mPFC, pSTS, TPJ and precuneus), separately for surface area and thickness across three waves (referred to as T1−T3). Figure 1, Panel A, shows an example LGM to model growth in mPFC surface area across three waves. LGMs are a highly flexible structural equation modelling (SEM) technique to examine what type of developmental patterns can best describe the data. Specifically, LGMs provide estimates of the mean intercept (e.g. the average baseline level of surface area and thickness obtained at the first assessment of the study) and mean level change across waves (referred to as the mean slope). In addition to these mean level intercept and slopes, LGMs can also examine whether individuals significantly vary around these mean level intercept and the rate of change (i.e. slopes). These individual differences in intercept and slope are captured by a variance component (referred to as a random slope). For our first aim, we examined the best fitting model to describe the data. That is, we tested an intercept only model, and a fixed and random linear and quadratic model. We compared these different models with the AIC (Akaike Information Criterion; Akaike, 1998) and BIC (Bayesian Information Criterion; Schwarz, 1978). The models with the lowest AIC and BIC values were preferred. If the AIC and BIC were inconsistent in their support for one model, we used the sample-size-adjusted BIC (Sclove, 1987) as an additional fit indicator to select the best fitting model.

To investigate our second aim, we extended the growth curve models to investigate whether individual differences in the intercept and linear and quadratic slopes predicted friendship quality at T3. Given our interest in the development and maintenance of high-quality friendships we controlled for the level of earlier friendship quality at T1 when predicting friendship quality at T3. In doing so, we could examine whether changes in friendship quality from T1 to T3 could be predicted by the baseline level and changes in social brain regions over time. In these growth models we controlled for possible gender differences in intercepts and slopes of the social brain regions. In addition, we included gender as a covariate of friendship quality at T3. If these additional age and gender covariates did not significantly (i.e. *P*-values < 0.05) predict intercept and slopes of social brain regions, they were omitted from the final models for reasons of model parsimony. Figure 1, Panel B, shows an example model of how intercept, linear and quadratic growth of mPFC surface area predict change in friendship quality.

Due to the accelerated longitudinal design of the Braintime study, participants varied significantly in age at study inclusion. See online Supplementary Figure S2 for the age distribution across time points. To account for this age heterogeneity at each wave, we applied the TSCORES option in ‘Mplus’ to scale the factor loadings for each participant based on his or her actual age at each measurement. In short, this modelling approach allows each participant to contribute to the estimation of parts of the growth curve for which he or she has data. Please find a detailed description of the TSCORES option modelling procedure as online Supplementary material S2. See Mehta and West (2000) for a detailed discussion of modelling age heterogeneity in latent growth models.

## Results

Means, standard deviations and correlations between study variables can be found as online Supplementary Table S1.

### Social brain development

#### Mean level development.

**Fig. 2.**
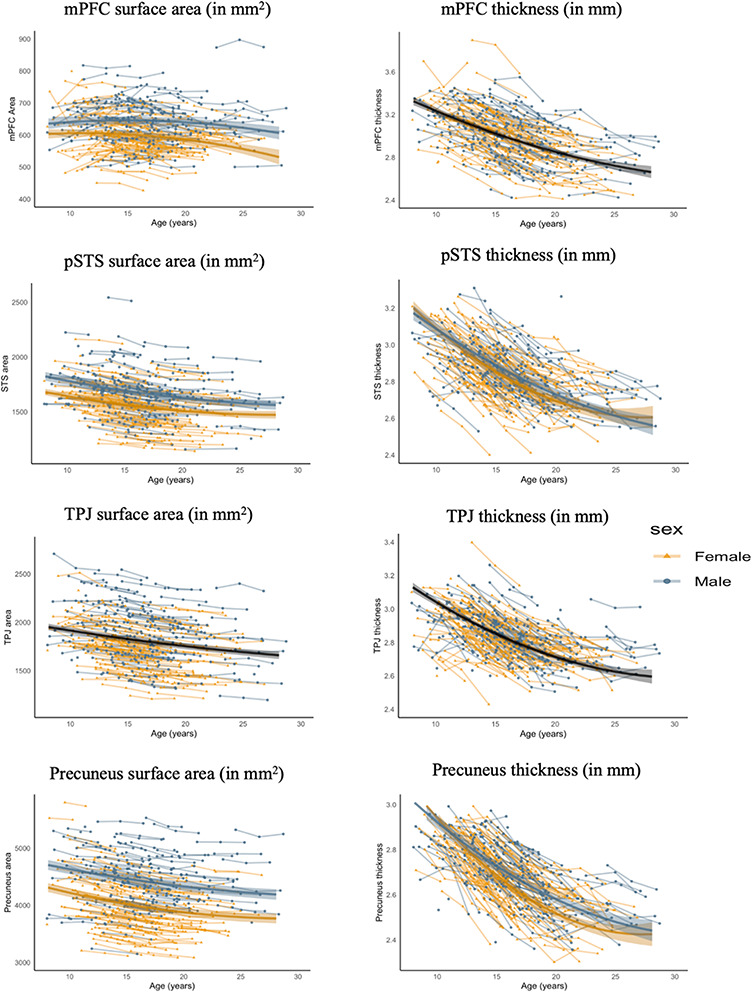
Observed individual trajectories for each region of interest. mPFC = medial prefrontal cortex; pSTS = posterior superior temporal sulcus; TPJ = temporoparietal junction. Estimated population trajectories for each gender are shown by coloured lines. If no gender differences were present, we plotted the average developmental trajectory across gender in solid black lines. The shaded areas represent the 95% confidence intervals.

We first tested for mean level changes in social brain regions, using latent growth curve models. For all social brain regions (and for surface area and thickness) a quadratic model including random linear and random quadratic slopes provided the best fit to the data (Supplementary Table S2 shows the fit indices AIC and BIC for the different models). Figure 2 shows the raw individual trajectories and the mean developmental trajectories for the entire sample. Table 1 shows the mean level growth parameter estimates as well as the individual differences (referred to with σ^2^) around these mean level intercepts and slopes. Contrary to our hypotheses, mPFC area was relatively stable in early to middle adolescence, followed by a decrease over time into young adulthood. Consistent with our hypotheses, all the other social brain regions revealed a linear decrease that levelled off towards the end of adolescence into young adulthood.

**Table 1.** Growth factor estimates of social brain regions

		Growth factors and variance components				
	Mean int. (SE)	σ^2^	Mean LS (SE)	σ^2^	Mean QS (SE)	σ^2^
***mPFC***						
mPFC SA (in mm^2^)	6.16 (0.20)***	3.18***	0.31 (0.23)	3.68**	−0.17 (0.06)*	0.24**
mPFC thickness (in mm)	3.90 (0.07)***	0.30***	−0.77 (0.08)***	0.33**	0.12 (0.02)***	0.02*
***pSTS***						
pSTS SA (in mm^2^)	19.50 (0.23)***	6.84***	−2.92 (0.20)***	1.98***	0.49 (0.06)***	0.08***
pSTS thickness (in mm)	3.80 (0.06)***	0.18*	−0.85 (0.07)***	0.17*	0.15 (0.02)***	0.01
***TPJ***						
TPJ SA (in mm^2^)	21.78 (0.33)***	13.40***	−2.94 (0.33)***	7.02**	0.40 (0.09)***	0.46**
TPJ thickness (in mm)	3.72 (0.06)***	0.27*	−0.80 (0.07)***	0.27	0.15 (0.02)***	0.02
***Precuneus***						
Prec. SA (in mm^2^)	4.96 (0.06)***	0.40***	−0.65 (0.05)***	0.06	0.11 (0.02)***	0.00
Prec. thickness (in mm)	3.65 (0.06)***	0.10	−0.87 (0.06)***	0.12	0.16 (0.02)***	0.01

Int = intercept; LS = linear slope; QS = quadratic slope; mPFC = medial prefrontal cortex; pSTS = posterior superior temporal sulcus; TPJ = temporal parietal junction; Prec. = precuneus; SA = surface Area.

^*^ *P < *0.05.

^**^ *P *< 0.01.

^***^ *P *< 0.001.

#### Individual differences in social brain development.

Next, we tested for individual differences in intercept and rate of change in social brain development. Results revealed individual differences in social brain development (Table 1 for the mean level intercept and growth parameters and individual differences in intercept, linear slope and quadratic slope). First, the intercepts of all the social brain regions varied significantly across individuals, except for precuneus thickness. Second, three out of the four social brain regions revealed individual differences in the rate of linear and quadratic within-person changes over time. That is, mPFC (surface area and thickness), pSTS (surface area and thickness) and TPJ (only surface area) showed significant variability between persons in the rate of linear changes. No significant individual differences in change of precuneus surface area and thickness were found. In all models, except for TPJ thickness and precuneus thickness, the intercept correlated negatively with the linear slope (all *P*-values < 0.035), indicating that those individuals who started with a higher intercept showed a steeper decline in surface area and thickness over time in the respective social brain regions.

Adding gender as a covariate of the intercept and slopes of the social brain regions revealed that the linear slope of mPFC surface area was larger for boys, compared to girls, *b *= 0.87, *P* = 0.031. Differences in intercept and quadratic slope of mPFC surface area between boys and girls could not be determined with 95% confidence (i.e. all *P*-values > 0.064).

Differences in intercept and slopes of mPFC thickness between boys and girls could not be determined with 95% confidence (all *P*-values > 0.39). Concerning pSTS surface area, boys showed a higher intercept compared to girls, *b *= 1.83, *P *= 0.001, but the linear and quadratic slopes did not differ between boys and girls (all *P*-values > 0.475). pSTS thickness intercept was lower for boys, *b *= −0.23, *P *= 0.020. In addition, boys showed a less steep linear decline in pSTS thickness, *b *= 0.31, *P *= 0.006, but a faster quadratic decrease, *b *= −0.09, *P *= 0.004, compared to girls. Differences in intercept and slopes for TPJ surface area between boys and girls could not be determined with 95% confidence (i.e. all *P*-values > 0.063). Concerning surface area of the precuneus, boys showed a higher intercept compared to girls, *b *= 0.32, *P *= 0.001, but no differences in linear and quadratic slopes (*P*-values* *> 0.084). The intercept of precuneus thickness was lower for boys, *b = *−0.30, *P *< 0.001, while the linear decline of precuneus thickness was less steep for boys, *b* *= *0.38, *P* < 0.001, and the quadratic slope was more negative for boys, *b *= −0.10, *P *= 0.001. Figure 2 shows the mean level trajectory differences in intercept and slopes between boys and girls.

### Individual differences in social brain development and friendship quality

Addressing our final aim, we examined whether individual differences in baseline and within-person changes in social brain regions over time predicted change in friendship quality (Figure 1, Panel B shows the estimated model for mPFC surface area as an example). Table 2 shows the parameter estimates of intercept and slopes predicting friendship quality.

**Table 2.** Unstandardized parameter estimates and standard errors of social brain regions predicting friendship quality at T3 controlling for friendship quality at T1^a^

		Friendship quality T3^b^	
Predictor		Parameter	SE
mPFC surface area (mm^2^)	Intercept	0.07*	0.03
	LS	−1.50**	0.49
	QS	−6.10**	1.82
mPFC thickness (mm)	Intercept	0.15*	0.06
	LS	0.48	0.96
	QS	1.63	3.64
pSTS surface area (mm^2^)	Intercept	−0.01	0.01
	LS	0.02	0.04
	QS	0.07	0.19
pSTS thickness (mm)	Intercept	−0.00	0.03
	LS	−0.04	0.15
	QS	Na	Na
TPJ surface area (mm^2^)	Intercept	−0.00	0.01
	LS	0.01	0.03
	QS	0.06	0.08
TPJ thickness (mm)	Intercept	−0.11***	0.03
	LS	−0.11	0.10
	QS	Na	Na
Prec. surface area (mm^2^)	Intercept	0.05	0.06
	LS	0.15	0.47
	QS	Na	Na
Prec. thickness (mm)	Intercept	0.05	0.03
	LS	−0.01	0.13
	QS	Na	Na

Na = Due to the non-significant variance between persons in the quadratic slope parameters, these models did not converge. We therefore fixed the quadratic slope variance to zero in these models. mPFC = medial prefrontal cortex; pSTS = posterior superior temporal sulcus; TPJ = temporal parietal junction; Prec. = Precuneus.

^a^ See online Supplementary material S3 for the exact *P*-values.

^b^ We controlled for T1 friendship quality.

^*^ *P* < 0.05.

^**^ *P* < 0.01.

^***^ *P* < 0.001.

#### mPFC.

Results revealed that those individuals with a higher intercept of mPFC surface area reported higher friendship quality at T3, above and beyond earlier levels of friendship quality at T1. However, individuals who showed a stronger linear increase in middle adolescence and a less steep subsequent decrease towards the end of adolescence (as modelled with the quadratic slope) reported lower friendship quality at T3. For mPFC thickness, a higher intercept predicted higher quality friendships over time. Individual differences in slopes for thickness of the mPFC did not significantly predict friendship quality.

#### TPJ.

When individuals showed a higher TPJ thickness intercept, they reported lower friendship quality over time, relative to individuals with a lower TPJ intercept. Surface area intercept and slopes did not predict friendship quality, longitudinally. Because individual differences in the quadratic slope were close to zero (See Table 1 for these parameter estimates), the model where the quadratic slope predicted friendship quality did not converge. We therefore fixed the quadratic slope variance to zero and did not include the quadratic slope as a predictor of friendship quality over time.

#### Precuneus.

Individual differences in precuneus surface area and thickness intercept and linear slopes did not significantly predict changes in friendship quality over time. Similar to TPJ thickness, the variance around the quadratic mean level slope of surface area and thickness was close to zero. As a result, a model including the quadratic slope as a predictor of friendship quality did not converge.

#### pSTS.

Similar to the precuneus, individual differences in intercept and linear slopes of pSTS surface area and thickness did not significantly predict changes in friendship quality over time. The model that included the quadratic slope of pSTS thickness as a predictor of friendship quality did not converge due to close to zero variance in the quadratic slope.

In sum, individual differences in the starting level (i.e. intercept) and rate of change in surface area and thickness of the mPFC and TPJ, but not pSTS and precuneus, predicted changes in friendship quality.

## Discussion

A commonly held assumption is that adolescents differ from each other in the structural development of the social brain (mPFC, pSTS, TPJ and precuneus; Mills *et al.*, 2014; Foulkes and Blakemore, 2018). Moreover, it is often hypothesized that these individual differences in brain development relate to individual differences in social behaviour over time (Burnett *et al.*, 2011; Blakemore, 2012). The present study empirically tested these assumptions, for the first time, in a large longitudinal brain imaging study.

First, we replicated previous findings on the group-level structural development of social brain regions. Specifically, surface area and thickness of all social brain regions decreased non-linearly from late childhood across adolescence, and into young adulthood (Mills *et al.*, 2014). These findings further substantiate an average developmental pattern of protracted social brain development from childhood into young adulthood (Mills *et al.*, 2014).

### Individual differences in social brain development

Importantly, however, our results confirmed prior speculations on the substantial individual differences around this average pattern of brain development (Foulkes and Blakemore, 2018). Specifically, we found that individuals differ from each other in both their initial level and the change rate at which their social brain matures, especially for surface area in the mPFC, pSTS and TPJ (see also Mills *et al.*, 2014). For cortical thickness, the observed changes in the pSTS (quadratic change) and TPJ (linear and quadratic change) were less variable between persons, compared to changes in surface area in these regions (which all showed significant between-person variability in the rate of change). Together, these findings provide the first empirical support that developmental changes in surface area are more likely to vary between individuals, compared to developmental changes of cortical thickness (Tamnes *et al.*, 2017; Mills and Tamnes, 2018).

In contrast to the mPFC, TPJ and pSTS, the rate of change of the precuneus (both surface area and cortical thickness) did not differ significantly between persons but showed a relatively consistent pattern of brain maturation across persons. Consistent with prior work, results did reveal individual differences in the starting levels (i.e. intercepts) of precuneus surface area (e.g. Vijayakumar *et al.*, 2016; Wierenga *et al.*, 2019). The relatively similar development of the precuneus across persons is consistent with recent findings from a twin study that showed that the structure of the precuneus is particularly genetically driven and less sensitive to environmental influences (van der Meulen *et al.*, 2020). Future work is needed to replicate our finding of relatively similar developmental trajectories of precuneus surface area and thickness across individuals.

### Individual differences in social brain development and friendship quality

The identification of heterogeneity in developmental change of social brain regions is an important first step (Foulkes and Blakemore, 2018). Yet, prior research highlights the importance of examining whether these individual differences in neurobiological developmental trajectories have predictive value for relevant outcomes (e.g. Rosenberg *et al.*, 2018). The social brain network is considered an important neurobiological predictor of social behaviours in adolescence (e.g. Blakemore, 2012). Therefore, we examined whether individual differences in intercept and the magnitude of change in the social brain predicted changes in friendship quality over time. Results revealed that higher baseline levels (i.e. intercepts) of surface area and cortical thickness of the mPFC predicted higher friendship quality at T3, while controlling for earlier levels of friendship quality at T1. These findings may suggest a developmental window of opportunity for social development that differs between individuals (Crone and Elzinga, 2015). Speculatively, a higher mPFC cortical thickness and surface area intercept may indicate higher levels of neural plasticity across adolescence, which might provide adolescents more opportunities to learn new social-cognitive skills (e.g. Blakemore and Mills, 2014).

Moreover, consistent with previous work (Ferschmann *et al.*, 2018, 2019), those individuals who showed a stronger decrease in mPFC surface area over time (reflecting advanced cortical thinning) developed higher quality relationships over time, while controlling for earlier levels of relationship quality. This finding supports the predictive specificity of change in mPFC surface area as a predictor of change in friendship quality above and beyond earlier levels of friendship quality.

In addition to the mPFC, individuals with a relatively high initial level of TPJ cortical thickness reported lower friendship quality over time. Thus, higher intercept levels of the mPFC and lower intercept levels of TPJ predicted higher friendship quality. These results indicate that lower TPJ starting levels possibly reflect more advanced brain maturation when predicting social functioning over time. Functional neuroimaging studies have revealed that increases in TPJ activity during a social decision-making task predicted higher levels of peer acceptance during adolescence (Will, Crone, Lier, and Güroğlu, 2018). Future studies should examine the role of TPJ levels in more detail when predicting adolescents’ social functioning.

Together, these findings suggest that structural levels and development of the mPFC and TPJ are specifically crucial for friendship quality development. Possibly, mPFC and TPJ functioning are specifically related to friendship quality, through their role in facilitating social-cognition capacities such as mentalizing and other orientation. Prior studies also emphasized the important role of the mPFC in self- and other-related thinking (Crone and Fuligni, 2020) and of the TPJ in intentionality understanding (Güroğlu, van den Bos, van Dijk, Rombouts, and Crone, 2011) and prosocial behaviours for friends (Schreuders, Klapwijk, Will, Güroğlu, 2018). Individual differences in the level and change of surface area and cortical thickness of the pSTS and precuneus did not significantly predict friendship quality. Thus, even though social brain development is often interpreted as a general network, different subregions within the social brain network may contribute to the development of different social behaviours in different ways.

What mechanisms might account for the observed linkages between accelerated social brain maturation of mPFC surface area and the development of high-quality friendship relationships? Accelerated cortical thinning is considered to mirror increasing regional specialization or fine-tuning within neural circuits across development, including the mPFC (Durston *et al.*, 2006; Johnson *et al.*, 2009; Burnett *et al.*, 2011). Consistent with this notion of fine-tuning, functional imaging studies on mPFC activity during mentalizing tasks reported an age-related decrease in mPFC activity (Blakemore and Mills, 2014), which may also reflect this pattern of increased regional specialization or increased efficiency of processing mental states within integrated neural circuits. Future studies are needed that combine functional and structural MRI into one longitudinal design to test this hypothesis directly (see, Burnett *et al.*, 2011 for an in-depth discussion on the possible mechanisms linking neuroanatomy and functional activity).

We found that the intercepts of mPFC and TPJ cortical thickness but not the slopes significantly predicted friendship quality. For surface area, between-person variability in the slopes of mPFC predicted friendship quality. These findings suggest differential contributions of thickness and surface area in explaining individual differences in social functioning. Across the board, cortical thickness of the social brain showed less between-person variability in slopes compared to surface area. Although speculative, those brain regions that show more variability between individuals across age (as was the case for surface area in our study) might be more malleable to environmental input than others, and have more impact on social behavioural functioning as well. If change is more constant across individuals over time (as was the case for cortical thickness), especially existing individual differences in starting levels have most predictive power (Walhovd *et al.*, 2016), including the prediction of social behavioural outcomes.

### Strengths, limitations and future directions

The present study had several strengths. First, our relatively large sample and longitudinal study design allowed us to directly examine (a) individual differences in baseline levels (i.e. intercepts) and changes (i.e. slopes) in social brain regions and (b) predicting changes in friendship quality over time. Second, we controlled for earlier levels of friendship quality. In doing so, we were able to examine whether social brain development predicted unique changes in friendship quality above and beyond earlier friendship quality levels. The current study also had some limitations. First, we were limited in our assessment of the quality of relationships with each participant’s best friend. Future studies are needed that also examine the possible parallel changes in social cognitive strategies such as mentalizing (representing one’s own and others’ mental states), which are proposed to facilitate friendship quality over time (Frith and Frith, 2001). Second, we did not control our analyses for multiple testing. Third, we considered the development of the social brain as an independent variable that predicts social functioning. However, early childhood predictors of later social functioning, such as parental sensitivity (Raby *et al.*, 2015) have been found to predict later structural brain development as well (Kok *et al.*, 2015). Possibly, changes in the social brain may mediate these longitudinal linkages between parental sensitivity and social functioning across adolescence and young adulthood. Fourth, the current study examined whether intercept and slopes showed significant differences between persons. Yet, it is important to keep in mind that even if *P*-values of the social brain intercept and slope variances were not significant they can still significantly predict outcomes. Fifth, the sample was relatively homogeneous in terms of social-economic status. Future studies are needed to examine these relations in more detail.

## Conclusion

This study demonstrated, for the first time, individual differences in the level and magnitude of changes in the social brain from late childhood into young adulthood. Thereby, findings further substantiate the plasticity of the brain beyond childhood well into the third decade of life. Moreover, variation in the magnitude of brain maturation uniquely predicted changes in relationship quality with the best friend. Together, these findings illustrate the importance of moving beyond averages when studying brain development to predict social outcomes.

## Supplementary Material

nsaa166_Supp
