# Supplementary material for: Beyond the average brain: individual differences in social brain development are associated with friendship quality
Source: Soc Cogn Affect Neurosci. 2020 Dec 5;16(3):292–301. doi: 10.1093/scan/nsaa166 (PMC7943358; doi:10.1093/scan/nsaa166)
Supplement: nsaa166_Supp [file nsaa166_supp.zip › scan-20-195-File006.docx]

Online supplementary material S.1

The same procedure was uses as described in Becht et al. (2018). Tesla Philips Achieva MRI system, with a standard whole-head coil (Philips, Best, The Netherlands) at Leiden University Medical Centre. High-resolution T1-weighted anatomical scans were acquired (TR=9.8 ms, TE = 4.6 ms, flip angle = 8°, 140 slices, 0.875 mm x 0.875 mm x 1.2 mm, and FOV = 224 x 177 x 168 mm). Scan time for the anatomical scan was 296 s. A radiologist evaluated all T1 scans and no anomalous findings were reported. With respect to image processing, cortical reconstruction was performed with the longitudinal stream (Reuter et al., 2012) in FreeSurfer 6.0.0, a program for cortical surface reconstruction and volumetric segmentation (<http://surfer.nmr.mgh.harvard.edu/>). The procedure and technical details are described elsewhere (Fischl, Sereno, & Dale, 1999a; Fischl, Sereno, Tootell, & Dale, 1999b; Reuter, Schmansky, Rosas, & Fischl, 2012). To extract reliable volume estimates, an unbiased within-subject template space and image (Reuter & Fischl, 2011) is created using robust inverse consistent registration (Reuter et al., 2010). Several processing steps, such as skull stripping, Talairach transformation, atlas registration as well as spherical surface maps and parcellations are then initialized with common information from the within-subject template, significantly increasing reliability and statistical power (Reuter et al., 2012).

Parcellation of the cortex into gyral regions was based on the Desikan-Killiany-Tourville atlas (Klein & Tourville, 2012). This labelling process involved surface inflation (Fischl et al., 1999a) and registration to a spherical atlas based on subject specific cortical folding patterns (Fischl et al., 2004a, b).

Online supplementary material S.2

Because adolescents varied significantly in age at each measurement wave, we applied a model where individually varying times of observations could be estimated (i.e., TSCORES option in Mplus, Muthén & Muthén, 1998-2012). In conventional LGM, it is assumed that data are collected at an identical set of fixed ages for all individuals (Mehta & West, 2000). In these models, slope factor loadings of a linear slope are fixed across all individuals to be 0, 1, 2, for Waves 1-3, respectively. However, with the TSCORES option the LGM takes into account heterogeneity in age at each measurement wave. That is, rather than defining linear growth with fixed factor loadings, age is now included in the model as a defining variable to scale the factor loadings and estimate the growth curve. Consider the example where we put the intercept at age 12 years. In this example, the starting point of the developmental trajectory is set at age 12 by fixing the factor loading for each observed measure for each individual to reflect deviation in years from age 12. Therefore, because adolescents vary in age at each measurement wave, the factor loadings are unique to each individual. For instance, an adolescent with an actual age of 12 years at the first measurement wave will obtain the linear slope factor loadings of 0, 1, and 2 for age 12, 13, and 14 years, respectively. However, an adolescent aged 15 years at the first measurement wave will obtain linear slope factor loadings of 3, 4, and, 5 for age 15, 16 and 17 years, respectively. Thus, each individual contributes to the estimation of parts of the growth trajectory for ages at which he or she does provide data (please see Mehta & West, 2000 for a detailed discussion of modelling individually varying times of observations).

Table S.1

*Means, standard deviations, and correlations among study variables*


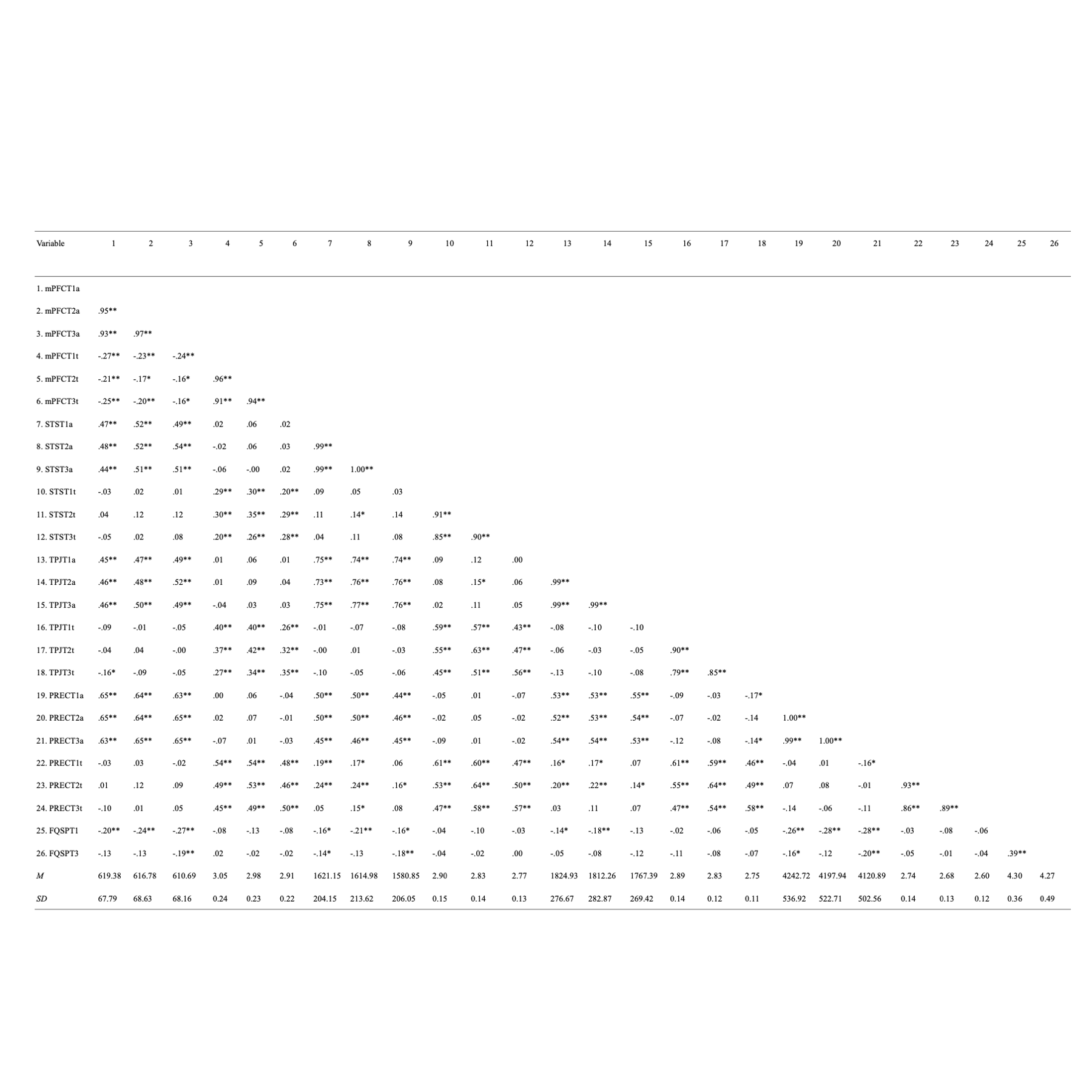

*Note.* a = Surface area (in mm^2^); t = thickness (in mm); FQSPT1 = friendship quality T1; FQSPT3 = friendship quality T3.
* *p* < .05. ** *p* < .01.

| Table S.2 |  |  | |  | |  |  | | |  | |  |  | | |  | |  |  |  |  |  |  |  |
| --- | --- | --- | --- | --- | --- | --- | --- | --- | --- | --- | --- | --- | --- | --- | --- | --- | --- | --- | --- | --- | --- | --- | --- | --- |
| *Fit indices of the latent growth curve models for all social brain regions* | | | | | | | | | | | | | | | | |  | |  |  |  |  |  |  |
| **Social brain** Intercept only | | | | | | | |  | Linear fixed | | | | |  | linear random | | | |  | quadratic fixed | |  | quadratic random | |
| **region** | | | AIC | | BIC | | |  | AIC | | BIC | | |  | AIC | | BIC | |  | AIC | BIC |  | AIC | BIC |
| mPFC SA | | | 446.76 | | 457.54 | | |  | 421.96 | | 436.33 | | |  | 407.13 | | 428.70 | |  | 387.31 | 412.47 |  | **357.45** | **393.40** |
| mPFC Thick | | | -568.97 | | -558.18 | | |  | -993.79 | | -979.42 | | |  | -1007.65 | | -986.08 | |  | -1016.80 | -991.64 |  | **-1042.62** | **-1006.67** |
| STS SA | | | 1609.70 | | 1620.48 | | |  | 1130.88 | | 1145.25 | | |  | 1075.01 | | 1096.57 | |  | 1036.11 | 1061.28 |  | **1028.35** | **1064.30^1^** |
| STS Thick | | | -898.92 | | -888.13 | | |  | -1396.97 | | -1382.59 | | |  | -1430.84 | | -1409.27 | |  | -1473.94 | -1448.78 |  | **-1495.28** | **-1459.34** |
| TPJ SA | | | 1934.77 | | 1945.56 | | |  | 1536.64 | | 1551.01 | | |  | 1528.94 | | 1550.51 | |  | 1522.7 | 1547.86 |  | **1481.633** | **1517.58** |
| TPJ Thick | | | -993.30 | | -982.51 | | |  | 1404.69 | | -1390.31 | | |  | 1457.16 | | -1435.59 | |  | -1480.43 | -1455.27 |  | **-1511.03** | **-1475.08** |
| Prec SA | | | -376.25 | | -365.46 | | |  | -922.39 | | -908.01 | | |  | -950.14 | | -928.57 | |  | -1006.38 | -981.22 |  | **-1013.36** | **-977.42^1^** |
| Prec Thick | | | -934.11 | | -923.32 | | |  | -1545.26 | | -1530.88 | | |  | -1579.02 | | -1557.46 | |  | -1646.30 | -1621.14 |  | **-1669.57** | **-1633.62** |

*Note.* Preferred final models are depicted in bold. AIC=Aikaike information criterion; BIC= Bayesian information criterion; mPFC=medial prefrontal cortex; STS=superior temporal sulcus; TPJ=temporal parietal junction; Prec.= Precuneus; SA=Surface area; Thick = thickness.

^1^  = In this case AIC and BIC were inconsistent in their support for one model. Therefore, we used the sample-size adjusted BIC (ssaBIC) as an additional criterion to select the best fitting model. For both the STS SA and Precuneus SA the random quadratic model was favoured.

| Table S.3  *Unstandardized parameter estimates and standard errors of  social brain regions predicting friendship quality at T3 controlling for friendship quality at T1, including p-values* | | | | |  |
| --- | --- | --- | --- | --- | --- |
|  |  | Friendship quality T3^1^ | | | *p-value* |
| Predictor |  |  | Parameter | SE |  |
| **mPFC surface area (mm^2^)** | Intercept |  | 0.07* | 0.03 | .014 |
|  | LS | - | 1.50** | 0.49 | .002 |
|  | QS | - | 6.10** | 1.82 | .001 |
| **mPFC thickness (mm)** | Intercept |  | 0.15* | 0.06 | .020 |
|  | LS |  | 0.48 | 0.96 | .615 |
|  | QS |  | 1.63 | 3.64 | .654 |
| **pSTS surface area (mm^2^)** | Intercept | - | 0.01 | 0.01 | .688 |
|  | LS |  | 0.02 | 0.04 | .691 |
|  | QS |  | 0.07 | 0.19 | .712 |
| **pSTS thickness (mm)** | Intercept | - | 0.00 | 0.03 | .975 |
|  | LS | - | 0.04 | 0.15 | .782 |
|  | QS |  | Na | Na | Na |
| **TPJ surface area (mm^2^)** | Intercept | - | 0.00 | 0.01 | .872 |
|  | LS |  | 0.01 | 0.03 | .802 |
|  | QS |  | 0.06 | 0.08 | .430 |
| **TPJ thickness (mm)** | Intercept | - | 0.11*** | 0.03 | .000 |
|  | LS | - | 0.11 | 0.10 | .277 |
|  | QS |  | Na | Na | Na |
| **Prec. surface area (mm^2^)** | Intercept |  | 0.05 | 0.06 | .424 |
|  | LS |  | 0.15 | 0.47 | .750 |
|  | QS |  | Na | Na | Na |
| **Prec. thickness (mm)** | Intercept |  | 0.05 | 0.03 | .100 |
|  | LS | - | 0.01 | 0.13 | .968 |
|  | QS |  | Na | Na | Na |

*Note.* ^1^ = We controlled for T1 friendship quality. Na = Due to the
non-significant variance between persons in the quadratic slope parameters,
these models did not converge. We therefore fixed the quadratic slope
variance to zero in these models. mPFC = medial prefrontal cortex;
pSTS = posterior superior temporal sulcus; TPJ = temporal parietal junction;
Prec. = Precuneus; *p < .05. **p < .01. ***p < .001.

*
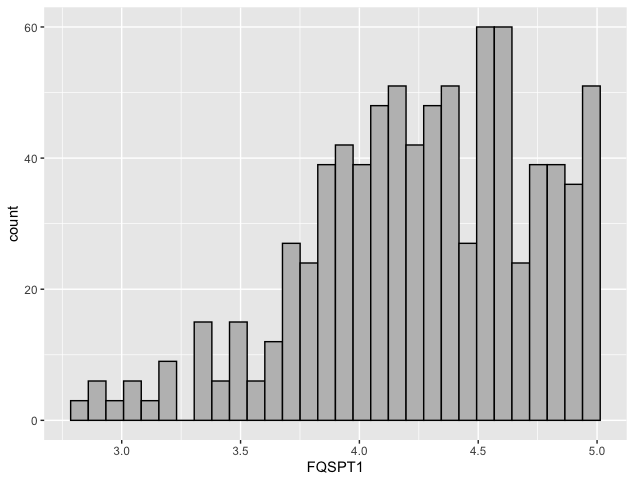
*

**A**

**B)**

*
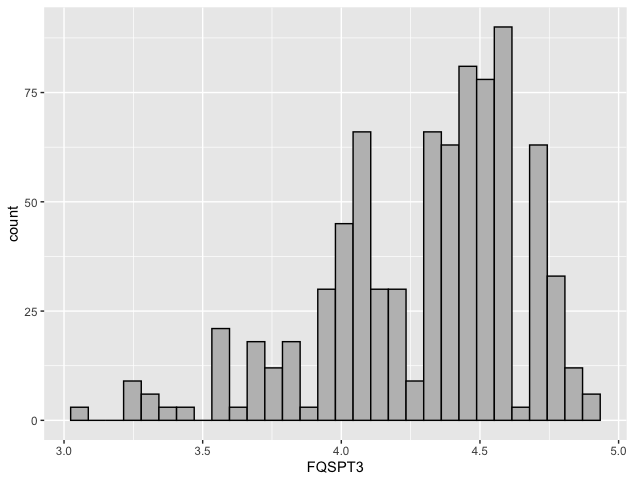
*

*Figure S.1.* Histograms of the mean friendship quality scores at T1 (panel A) and T3 (panel B). *Note:* FQSPT1 = friendship quality T1; FQSPT3 = friendship quality T3.


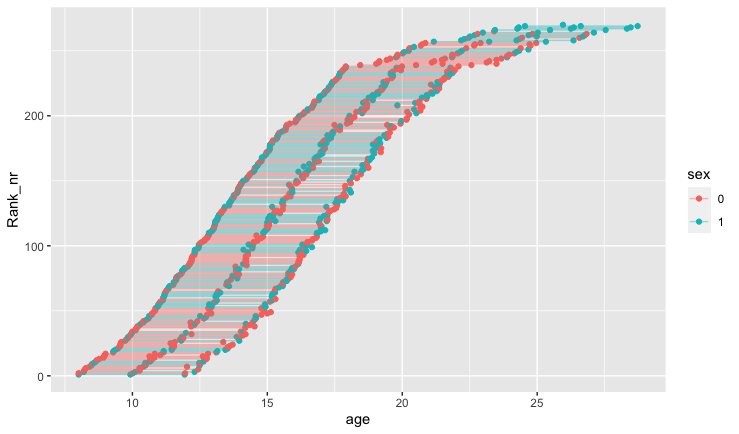


Male

Female

*Figure S.2.* Age distribution of the accelerated longitudinal Braintime study.
